# Multiple Resistance Evolution in Bipyridylium-Resistant *Epilobium ciliatum* After Recurrent Selection

**DOI:** 10.3389/fpls.2018.00695

**Published:** 2018-05-28

**Authors:** Berhoz K. Tahmasebi, Ricardo Alcántara-de la Cruz, Esteban Alcántara, Joel Torra, José A. Domínguez-Valenzuela, Hugo E. Cruz-Hipólito, Antonia M. Rojano-Delgado, Rafael De Prado

**Affiliations:** ^1^Department of Agronomy and Plant Breeding, University of Mohaghegh Ardabili, Ardabil, Iran; ^2^Departamento de Entomologia/BIOAGRO, Universidade Federal de Viçosa, Viçosa, Brazil; ^3^Departamento de Agronomía, Universidad de Córdoba, Córdoba, Spain; ^4^Departament d'Hortofructicultura, Botànica i Jardineria, Agrotecnio, Universitat de Lleida, Lleida, Spain; ^5^Department of Agricultural Parasitology, Chapingo Autonomous University, Chapingo, Mexico; ^6^Bayer CropScience Mexico, Mexico City, Mexico; ^7^Department of Agricultural Chemistry and Edaphology, University of Cordoba, Cordoba, Spain

**Keywords:** 5-enolpyruvylshikimate-3-phosphate synthase, acetolactate synthase, glutamine synthetase, fringed willowherb, photosystem I, protoporphyrinogen oxidase, synthetic auxins

## Abstract

The use of herbicides with different modes of action is the primary strategy used to control weeds possessing resistance to a single mechanism of action (MOA). However, this practice can lead to selection for generalist resistance mechanisms and may cause resistance to all MOAs. In this research, we characterized the resistance to diquat/paraquat (bipyridiliums) in an *Epilobium ciliatum* biotype (R1) collected in an olive orchard from Chile, where alternatives herbicides (2,4-D, glyphosate, glufosinate, flazasulfuron and pyraflufen-ethyl) with different MOAs were used, but they have also showed failure in controlling this species. Because the resistance/susceptibility patterns of the R1 biotype to glufosinate, 2,4-D and pyraflufen-ethyl were not clear, a recurrent resistance selection was carried out in field and greenhouse using these herbicides on R1 plants for three generations (R2 biotype). One biotype that was never treated with herbicides (S) was included as control. Results indicated that the S biotype was controlled at the field dose of all herbicides tested. The biotype R1 exhibited resistance to diquat, paraquat and flazasulfuron and natural tolerance to glyphosate. The R2 biotype displayed resistance to glufosinate, 2,4-D and pyraflufen-ethyl with LD_50_ (herbicide dose to kill 50% of plants) values higher than field doses in all assays. Physiological and biochemical studies determined the resistance to diquat of the R1 biotype, which was due to impaired translocation. The resistance to flazasulfuron in the R1 and R2 biotypes was confirmed by the low sensitivity of the acetolactate synthase (ALS) activity compared to the S biotype. The similar accumulation of shikimate in treated S, R1, and R2 plants with glyphosate supported the existence of innate tolerance to this herbicide in *E. ciliatum*. Resistance to glufosinate, 2,4-D and pyraflufen-ethyl in the R2 biotype, acquired after recurrent selection, was determined by low sensitivity of the glutamine synthetase, low accumulation of ethylene and protoporphyrinogen IX oxidase, respectively, in comparison to the S biotype. *Epilobium ciliatum* from Chilean olive orchards had resistance to only two MAOs (photosystem I and ALS inhibitors), but resistance to five MOAs could occur in the next cropping seasons, if alternatives to weed management, other than herbicides, are not included.

## Introduction

*Epilobium ciliatum* Raf. (fringed willowherb or American willowherb) is a troublesome annual weed belonging to Onagraceae, known as the willowherb or evening primrose family that contains approximately 170 species. This family is exceptional for its morphological, ecological and cytological variety (Myerscough and Whitehead, 1966). *Epilobium ciliatum* was first recorded in Britain in 1891 and was then spread globally. This species has a wide ecological niche and can produce thousands of seeds in a season. Its growth can be found in areas at sea level or 3,000 m above sea level in dry soils, open or disturbed woodlands, grasslands, and along roadsides (Myerscough and Whitehead, 1967).

*Epilobium ciliatum* is normally controlled with post-emergence application of paraquat and/or diquat (PS I electron diverter, also known as bipyridyliums) in perennial crops such as nurseries, orchards and hops in Northern Europe (Bulcke et al., 1987). Although paraquat is one of the most toxic herbicides used in the last 60 years, it is used extensively in approximately 100 countries on more than 100 crops without restrictions. Paraquat is a non-selective post-emergence herbicide that causes peroxidative stress in plants (Vartak and Bhargava, 1999).

The repeated application of bipyridylium herbicides can easily cause selection of resistant and tolerant weed biotypes (Hawkes, 2014). Both herbicide resistance and tolerance implies that there was no selection or genetic manipulations that allowed survival of weeds. To date, 32 species have evolved bipyridylium resistance globally, and *E. ciliatum* was detected as being resistant to paraquat in Belgium and Unite Kingdom in 1982 and 1989, respectively (Heap, 2018). Usually resistance to these herbicides is associated with reduced translocation of bipyridylium out of treated tissues (Moretti and Hanson, 2017). Additional reports indicated that protective enzymes also confer paraquat resistance (Hawkes, 2014).

The occurrence of bipyridylium resistance has led to an increase in the use of the herbicides glyphosate [5-enolpyruvylshikimate-3-phosphate synthase (EPSPS) inhibitor], glufosinate [glutamine synthetase (GS) inhibitor], 2,4-D (synthetic auxin), flazasulfuron [acetolactate synthase (ALS) inhibitor], and pyraflufen-ethyl [protoporphyrinogen oxidase (PPO) inhibitor] among others. Using numerous herbicides allows a broad herbicidal spectrum that includes activity against monocotyledonous and dicotyledonous weeds, and rapid onset of action and long persistence of some herbicides (Ganie and Jhala, 2017).

The use of herbicides with different mechanism of action (MOA), alone or in mixture, is the main tool to combat resistance to a specific group of herbicides (Tornisielo et al., 2013). The proper implementation of this practice can lead to selection of generalist target or non-target site resistance mechanisms, inducing the evolution of weed biotypes resistant to multiple MOAs (Neve and Powles, 2005). In cases of resistance multiple to herbicides, weeds evolved from monogenetic to polygenetic resistance (Heap, 2014). Many plants, particularly weeds, can reach this condition, because they contain a tremendous amount of genetic variation that allows them to survive under different biotic and abiotic conditions (Neve et al., 2009).

Since the early 1990s, herbicides have been the main tool for weed control in Chile (Valverde, 2007). In some cases, herbicides with different MOAs were applied widely to control bipyridylium-resistant *E. ciliatum* in olive trees in Chile; however, some of them have also shown failure in controlling this species. Here, we investigated the resistance to bipyridylium and the evolution of multiple resistance in *E. ciliatum* biotypes harvested in the Lolol province in Chile.

## Materials and methods

### Chemicals

Commercially formulated diquat, paraquat, glyphosate, glufosinate, flazasulfuron, 2,4-D and pyraflufen-ethyl (Table 1), were used for spraying *E. ciliatum* plants. Analytical grade (>99.5%) was used to determine the herbicide effects on physiological and biochemical studies in plants.

**Table 1:** Herbicides, formulation type (FT), percentage of concentration (PC), WSSA/HRAC group (Group), mechanism of action (MOA), field doses in g ai ha^−1^ (Dose), doses used in the curve dose-response in g ai ha^−1^ (Dose-response) and application time (Time) evaluated on the bipyridylium-resistant *Epilobium ciliatum* biotypes from Chile.

**Herbicide**	**Trade name (FT-PC)^a^, Manufacturer**	**Group^b^**	**MOA^c^**	**Dose**	**Dose-response**	**Application time^d^**
Diquat	Reglone® (SL, 17% w/w), Syngenta	22/D	PSI	400	0, 12.5, 25, 50, 100, 250, 500, 1,000, and 2,000	POST
Paraquat	Paratex® (SL, 20% w/v), Aragonesas Agro (ADAMA)	22/D	PSI	500	0, 25, 50, 100, 500, 1,000, 2,000, 4,000, and 6,000	POST
Glyphosate^e^	Roundup® Energy (SC, 50.9% w/w), Monsanto	9/G	EPSPS	720	0, 100, 200, 400, 600, 800, 1,000, 2,000, and 4,000	POST
Glufosinate	Finale® (SL, 20% w/v), Bayer CropScience	10/H	GS	750	0, 12.5, 25, 50, 100, 250, 500, 1,000, and 2,000	POST
Flazasulfuron	Terafit® (WG, 25% w/w), Syngenta	2/B	ALS	50	0, 1.25, 2.5, 5, 10, 20, 40, 80, and 160	PRE and early POST
2,4-D	U46® D Complet (SL, 60% w/v), Nufarm	4/O	SA	800	0, 12.5, 25, 50, 100, 250, 500, 1,000, and 2,000	POST
Pyraflufen-ethyl	Gozai® (CE, 2.65% w/v), Belchim Crop Protection	14/E	PPO	5	0, 0.15, 0.3, 0.6, 1.2, 2.4, 6.0, 12, and 24	Early POST

a *FT: SL, soluble (liquid) concentrate; SC, suspension concentrate; WG, water dispersible granules, and EC, emulsifiable concentrate. PC: w/w = weight/weight or w/g = weight/volume. Mention of trade names in this publication is solely for providing specific information and does not imply their recommendation*.

b *WSSA, Weed Science Society of America and HRAC, Herbicide Resistance Action Committee*.

c *MOA: PSI, photosystem I inhibitor (electron diverter); EPSPS, 5-enolpyruvylshikimate-3-phosphate synthase inhibitor; GS, glutamine synthetase inhibitor; ALS, acetolactate synthase inhibitor; SA, synthetic auxins, and PPO, protoporphyrinogen oxidase inhibitor*.

d *POS, post-emergence and PRE, pre-emergence*.

e *Doses expressed as g acid equivalent (ae) ha^−1^ (50.9% potassium salt of glyphosate equals 450 g ae L^−1^)*.

### Plant material

*Epilobium ciliatum* mature seeds of a biotype that is resistant (R1) to diquat/paraquat were collected from an olive orchard in the Lolol province, Chile (34°44′07″S71°42′16″W) in 2014. This orchard field had been treated for more than 20 years using PSI inhibiting herbicides, but in recent years, other herbicides (from the ALS, EPSPS, GS, PPO inhibitors and synthetic auxins chemical groups) have been used as alternatives to the first one. Seeds of a susceptible (S) biotype were also collected in 2014 from a closed area in which herbicides had never been applied.

The seeds were germinated in Petri dishes containing filter paper that was moistened with distilled water. Petri dishes were placed in a growth chamber at 28/18°C (day/night) with a photoperiod of 16 h, 850 μmol m^−2^ s^−1^ photosynthetic photon flux, and 80% relative humidity. All seedlings were transplanted into pots (one plant per pot) containing sand/peat in a 1:1 (v/v) ratio, and placed in a greenhouse with a 16 h photoperiod.

### Dose response to herbicides

Experiments were conducted using eight replicates (individual plants) for each *E. ciliatum* biotype (R1 and S) at eight doses of paraquat and diquat, and including one set of non-treated plants as controls (Table 1). The herbicide doses were applied at two different growth stages: rosette (BBCH14-16) and 10 cm height (tillering, BBCH55-60) plants. Glyphosate, glufosinate, flazasulfuron, 2,4-D and pyraflufen-ethyl were only applied at the rosette stage with eight doses of each herbicide (Table 1). The herbicides were applied in a laboratory chamber (SBS-060 De Vries Manufacturing, Hollandale, MN, USA) equipped with 8002 flat fan nozzles delivering 200 L ha^−1^ at the height of 50 cm from plant level. Experiments with alternative herbicides were repeated including the biotype R2, obtained from recurrent selection for resistance, as will be described later.

Plant mortality (LD) and fresh weight reduction (GR) were measured 21 days after treatment (DAT). After estimating LD_50_ (herbicide dose required to kill by 50% a weed population) and GR_50_ (dose required to reduce shoot weight by 50% relative to non-treated plants) values using log-logistic models (Y = c+{(d–c)/[1+(x/g)^b^]} or Y = (d)/1+(x/g)^b^), the resistance factors (RF = R/S) were computed as R-to-S GR_50_ or LD_50_ ratios.

### Recurrent selection

Because the resistance/susceptibility patterns of the R1 biotype to 2,4-D, glufosinate and pyraflufen-ethyl were not clear in the first set of dose-response assays, a recurrent selection for resistance to these three herbicides was conducted.

For the first generation, two thousand seedlings of R1 biotype were transplanted into plots (2 × 5 m) in the experimental field at the University of Cordoba (Spain). When plants reached the rosette stage, they were treated with pyraflufen-ethyl (PPO inhibitor) at 5 g ai ha^−1^. Two weeks later the surviving plants were treated with 2,4-D (synthetic auxin) at 400 g ai ha^−1^. Finally, surviving plants that were 10 cm in height (tillering stage) were treated with glufosinate (GS inhibitor) at 750 g ai ha^−1^. The herbicides were applied using a Pulverex backpack sprayer with a T coupling for the wand equipped with four flat fan nozzles, calibrated to deliver 200 L ha^−1^ at a spraying pressure of 200 kpa.

The effect of pyraflufen-ethyl was very fast. Growth stopped and leaves exhibited burns 2 DAT. Resistant plants (5%) began to regrow producing leaves. During the first days after 2,4-D treatment the plants stopped growing and classical uncontrolled tissue growth and epinasty appeared, followed by growth inhibition and death. However, more than 20% of the total plants recovered their normal growth 14 DAT. Finally, treatment with glufosinate caused a rapid effect after the first DAT, with appearance of plant chlorosis and necrosis. Between 40 and 50% of plants finished their reproductive cycle and mature seeds with multiple resistance (to PS I + GS + PPO + synthetic auxins) were harvested and used for the next generations.

For the second generation, approximately 500 seedlings were transplanted into trays (40 × 100 × 15 cm) containing the same soil mixture and growth conditions described in Plan Material section. Approximately 80% of the treated plants finished their reproductive cycle. Six months later the third generation was initiated and plant survival was 95% in this generation. The F_3_ progeny, hereinafter are referred as R2 biotype, was included together with biotypes R1 and S to repeat the dose-response experiments, as described above, and to conduct the physiological and biochemical studies to characterize multiple resistance.

### Physiological and biochemical studies

Seedlings of *E. ciliatum* R1, R2, and S biotypes were transplanted into pots and grown in growth chambers under the same set of conditions described in Plant Material section.

#### Diquat

^14^C-diquat absorption and translocation were evaluated at 3, 6, 12, and 24 h after treatment (HAT). R1 and S plants were treated when reached the rosette stage with a solution of ^14^C-diquat (specific activity 6.2789 MBq/mg, American Radiolabeled Chemicals, Inc., Saint Louis, MO, USA) plus commercial diquat formulation. The solution applied contained ^14^C-diquat providing 0.834 kBq μL^−1^ at the final concentration of 100 g ia ha^−1^ of diquat in 300 L. One drop of 1 μL plant^−1^ of solution was applied on the adaxial surface of the second youngest fully expanded leaf. After treatment, the plants were maintained in the growth chamber during 12 h in the dark before light was initiated (Moretti and Hanson, 2017).

To determine the absorption, the ^14^C-diquat treated plants were harvested (at the previous times) and the treated leaves were washed three times separately with 1 mL of water to recover the non-absorbed ^14^C-diquat. The washing solution was mixed with 2 mL of scintillation liquid (Ultima Gold, Perkin-Elmer, BV BioScience Packard). Samples were reserved to analysis the radioactivity. To determine the translocation, the whole plants were carefully removed from the pot (at the previous times) and washed, mainly the roots. The plants were individually divided into treated leaf, remainder of the plant and root system. The samples were stored in flexible combustion cones (Perkin-Elmer, BV BioScience Packard, Groningen, Netherlands), dried in an oven at 60°C for 48 h. Later, the samples were combusted in a Packard Tri Carb 307 biological oxidizer (Packard Instrument Co., Downers Grove, IL, USA). The CO_2_ released from the combustion was captured in 18 mL of a mix of Carbo-Sorb E and Permafluor (1:1 v/v) (Perkin-Elmer, BV BioScience Packard, Groningen, Netherlands).

The radioactivity in washing solution and combustion samples was quantified by liquid scintillation spectrometry in a LS 6500 scintillation counter (Beckman Coulter Inc., Fullerton, CA, USA) during 5 min per sample, and the measurement was repeated again 24 h later. The radioactive values were used to calculate recovery percentage as: % ^14^C-diquat recovered = (kBq in treated leaf + kBq reminder plant + kBq in root system + kBq from washes/ kBq total applied) × 100. Experiments were arranged in a completely random design with five replicates per biotype at each time evaluated.

#### Glyphosate

The shikimic acid accumulation produced by glyphosate was studied in the R1, R2, and S biotypes of *E. ciliatum* according to Shaner et al. (2005). Leaf disks (5 mm diameter) were harvested from the youngest fully expanded leaves from a batch of 15 plants of each biotype at the rosette stage. The glyphosate concentrations used were: 0.1, 10, 50, 500, and 1,000 μM. Absorbance was measured using a DU-640 spectrophotometer (Beckman Instruments Inc., Fullerton, USA) at 380 nm. The experiments had a completely random design using three tissue samples of 50 mg from each *E. ciliatum* biotype per glyphosate concentration. Experiments were repeated twice and results were expressed in mg of shikimic acid per gram of fresh tissue. The amount of shikimic acid was determined by comparison to a set of standard samples of known shikimic concentrations used for plotting calibration curve.

#### Flazasulfuron

ALS activity was measured using the product acetolactate estimation after conversion by decarboxylation in the presence of acid to acetoin (Hatami et al., 2016). Two grams of young leaves of *E. ciliatum* R1, R2, and S biotypes were ground using extraction buffer (3 mL g^−1^). The de-salted protein extract was used in ALS enzyme assays, with flazasulfuron as representative of the ALS inhibitor herbicides. The herbicide concentrations used were: 0, 1, 5, 10, 50, 100, 500, 1,000, 2,000, and 3,000 μM. ALS activity was assayed colorimetrically (at 520 nm) by measuring acetoin production and expressing this as a percentage in respect to the control. The experiment was repeated at least three times from independent protein extractions and the I_50_ values (inhibition of the ALS enzyme by 50%) were estimated. The protein concentration of the crude extract was measured using the Bradford method (Bradford, 1976).

#### Glufosinate

The glutamine synthetase (GS) response to glufosinate was determined using crude protein extracts isolated from *E. ciliatum* R1, R2, and S leaves (rosette stage) as described by Rojano-Delgado et al. (2013). The glufosinate concentrations used were: 0, 1, 10, 50, 100, 500, 1,000, and 5,000 μM. The glufosinate concentration that reduced the GS activity by 50% (I_50_) was used to calculate the resistance values (RF) values. The total protein (nmol of glutamine mg^−1^ of protein h^−1^) was measured following the Bradford's method (Bradford, 1976). This experiment was repeated twice using three replications per herbicide concentration.

#### 2,4-D

Plants at the rosette stage were sprayed with 2,4-D solutions (0, 200, 400, 600, 800, and 1,000 g ai ha^−1^) as in dose response curves. Twenty-four HAT, seedlings were excised and 400 g shoot fresh weight were placed into a 10 mL syringe with 1 mL distilled water and sealed (De Prado et al., 2000). The syringes were placed in a dark incubator at 27°C for 4 h and 1 mL of the headspace gas was analyzed for ethylene (C_2_H_4_) by gas chromatography (Shimabukuro and Hoffer, 1996). The C_2_H_4_ was expressed as nanoliter per gram of fresh weight by hour. There were five replicates per treatment and the experiment was repeated twice.

#### Pyraflufen-ethyl

Protoporphyrin oxidase IX (Protox IX) levels were determined following the method proposed by Dayan et al. (2015). Approximately 0.1 g leaf-disks (4 mm-diameter) of R1, R2, and S biotypes were incubated in a Petri dishes containing 6 mL of 2% (w/v) sucrose, 1 mM 2-(N-morpholine) ethanesulfonic acid and 100 μM technical pyraflufen-ethyl for 20 h at 25°C in darkness. After incubation, leaf disks were homogenized and centrifuged (Fernandez-Moreno et al., 2017). The supernatants were concentrated and reconstituted in 1 mL of methanol and filtered through a 0.2 μm nylon syringe filter to clean the samples. The extracts were stored in opaque glass vials at 20°C. An aliquot of 50 μL was injected into the HPLC system (Beckman Coulter 126 Gold System, Fullerton, California, USA). Protox IX concentrations in the extracts were quantified using a calibration curve obtained from a Protox IX standard (Sigma Aldrich, St. Louis, Missouri, USA). The results were expressed as nanomoles per gram of fresh weight. Treatments were carried out three times, with three repetitions per treatment.

## Results

### Dose response to PS I inhibiting herbicides

**Figure 1:**
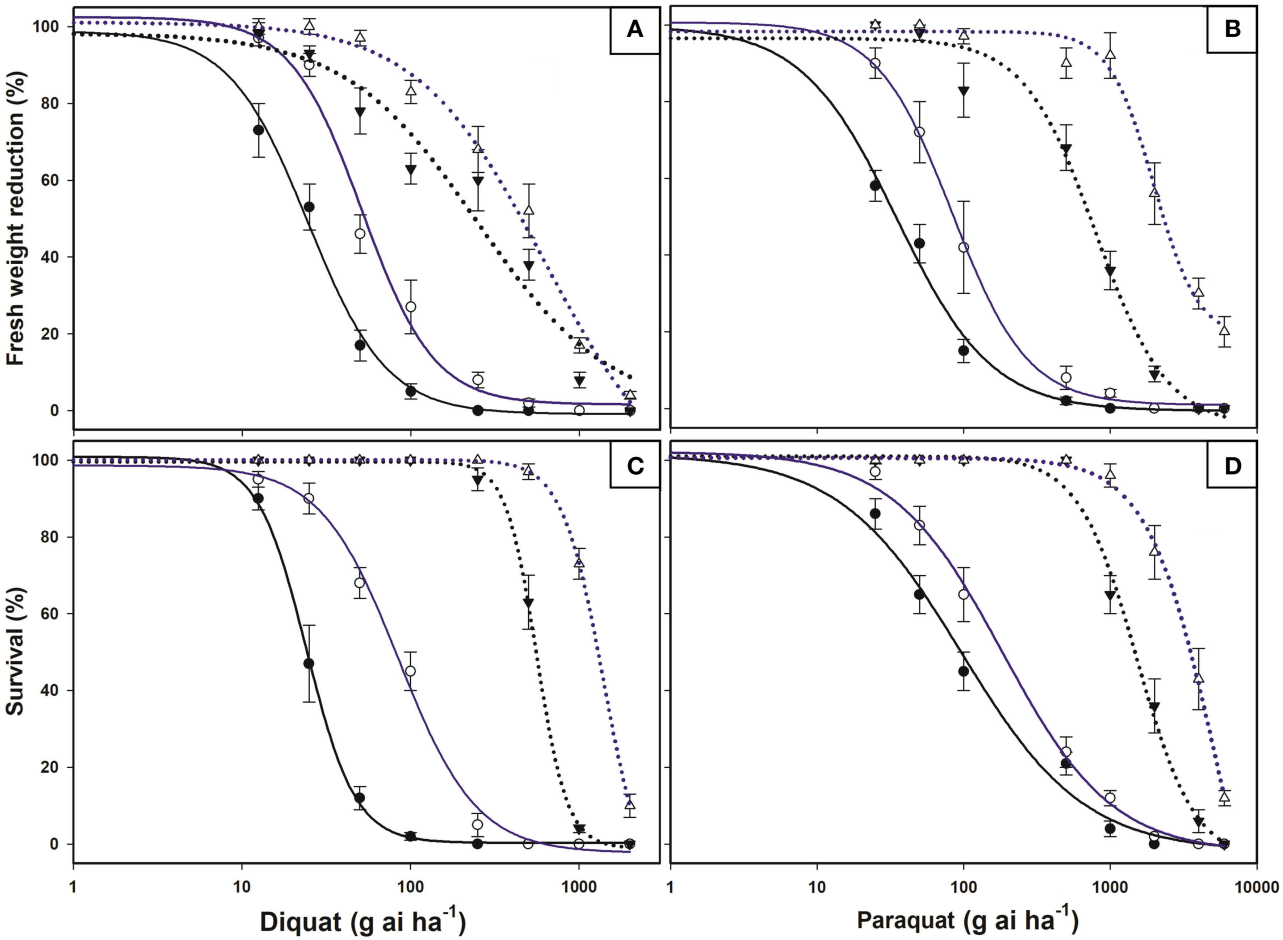
Log-logistic curves of bipyridylium-resistant *Epilobium ciliatum* S (solid lines) and R1 (dotted lines) biotypes, treated at the BBCH14-16 (black lines) and BBCH55-60 (blue lines) stages. Diquat **(A)** and Paraquat **(B)** dose-response curves based on the fresh weight reduction percentage with respect to untreated controls. Diquat **(C)** and Paraquat **(D)** dose-response curves of survival percentage with respect to untreated controls. Evaluations were conducted 21 days after treatments. Vertical bars are ± standard errors of the mean (*n* = 8).

Dose-response studies with diquat and paraquat on plants were concluded with 100% mortality in the *E. ciliatum* S biotype at doses well below the field doses. In contrast, the R1 biotype was markedly less affected by PS I inhibiting herbicides and required doses higher than those normally used by Chilean farmers in the Lolol area. The LD_50_ and GR_50_ values for R1 were higher than for S biotype (Figure 1, Table 2).

**Table 2:** LD_50_ and GR_50_ values of the R1 and S *Epilobium ciliatum* biotypes using diquat and paraquat at different growth stages (BBCH 14–16 and 55–60).

**Herbicide**	**BBCH**	**Biotype**	**GR_50_ ± SE**	**RF**	***c***	***d***	***b***	***R*^2^**	**LD_50_ ± SE**	**RF**	***c***	***d***	***b***	***R*^2^**
Diquat	14–16	S	24.6 ± 1.7	10.1	−0.86	98.78	1.86	0.99	37.2 ± 2.4	15.1	0.27	100.79	3.00	0.99
		R1	247.6 ± 31.6		0.0	98.27	1.11	0.93	560.2 ± 22.5		−1.03	99.56	4.76	0.99
	55–60	S	51.4 ± 5.2	12.1	1.57	102.42	2.04	0.98	84.9 ± 6.3	16.5	−0.28	98.60	1.87	0.99
		R1	619.6 ± 42.4		−3.31	101.11	1.15	0.98	1401.7 ± 37.6		−0.77	100.03	3.51	1.00
Paraquat	14–16	S	35.0 ± 3.0	22.7	−0.68	99.63	1.34	0.99	96.9 ± 17.4	15.7	−1.91	101.49	1.03	0.98
		R1	796.0 ± 48.4		−1.82	96.49	1.78	0.98	1521.2 ± 158.6		−0.65	101.02	2.18	0.98
	55–60	S	83.8 ± 7.8	23.7	0.91	100.66	1.69	0.99	184.1 ± 19.6	18.7	0.30	102.15	1.16	0.99
		R1	1986.6 ± 191.8		8.1	98.24	2.84	0.98	3447.4 ± 216.2		−1.71	100.54	1.85	0.99

*Data were pooled and fitted using the non-linear regression model: Y =c+{(d–c)/[1+(x/g)^b^]}, where Y is the is the fresh weight reduction or survival percentage; x (independent variable) the herbicide dose; c and d are the lower and upper limits, respectively; b is the slope of the curve; GR_50_ and LD_50_ expressed in g ai ha^−1^. ^b^RF = Resistance factor = GR_50_ or LD_50_ of the R biotype/GR_50_ or LD_50_ of the S biotype. ± Standard error of the mean (n = 8)*.

When both herbicides were applied in R1 and S plants before flowering (BBCH55-60 stage), herbicide efficacy decreased and resulted in a LD_50_ that was notably higher than younger plants (BBCH14-16 stage). The GR_50_ values also established high resistant factors (RF) to PS I inhibiting herbicides (Figure 1, Table 2).

### Response to alternative herbicides before and after recurrent resistance selection

For 20 years, *E. ciliatum* has been exposed to the PS I inhibiting herbicides (diquat/paraquat), and eventually herbicides with different MOAs (glyphosate, flazasulfuron, glufosinate, 2,4-D and pyraflufen-ethyl) were used as alternative. However, Chilean farmers have also reported failures in controlling *E. ciliatum* with these herbicides. Our results of the first set of dose-response assays were ultimately inconclusive to determine the status of resistance or susceptibility to glufosinate, 2,4-D and piraflufen-ethyl, because the GR_50_ and LD_50_ values of the R1 biotype were similar to the S biotype. R1 plants showed low resistance to glufosinate (FR = 2.9) and intermediate resistance to 2,4-D (FR = 3.9) and pyraflufen-ethyl (FR = 1.7) (Table 3). However, some individuals in the R1 biotype survived at doses equal to or higher than the field doses of these herbicides. For this reason, we conducted the recurrent selection for resistance in field and greenhouse during three generations, which gives origin to the R2 biotype. These experiments were aimed at testing if multiple resistance condition can occur in Chilean olive orchards in the next cropping seasons.

**Table 3:** LD_50_ and GR_50_ values of the R1, R2 and S *Epilobium ciliatum* biotypes using different herbicides, applied at the BBCH 14–16 growth stage.

**Herbicide**	**Biotype**	**GR_50_ ± SE**	**RF**	***d***	***b***	***R*^2^**	**LD_50_ ± SE**	**RF**	***d***	***b***	***R*^2^**
Glyphosate	R1	310.1 ± 23.1	1.1	102.06	1.42	0.98	931.8 ± 37.1	1.0	99.56	3.85	0.98
	R2	281.0 ± 28.8	1.0	104.11	1.40	0.99	989.1 ± 41.6	1.1	99.19	4.32	0.99
	S	270.9 ± 19.5	–	103.78	1.39	0.99	904.0 ± 58.0	–	94.63	3.22	0.97
Glufosinate	R1	60.4 ± 19.7	1.6	102.05	1.14	0.98	167.1 ± 22.8	2.9	100.18	3.91	0.99
	R2	269.8 ± 41.4	7.3	99.72	1.85	0.97	1096.1 ± 38.9	19.3	100.12	2.94	0.99
	S	36.9 ± 4.7	–	101.95	1.52	0.98	56.9 ± 3.4	–	101.66	1.73	0.98
Flazasulfuron	R1	37.4 ± 3.9	7.8	93.35	2.12	0.99	63.4 ± 6.6	3.4	100.31	4.73	0.99
	R2	41.7 ± 4.1	8.7	95.88	2.33	0.98	66.5 ± 4.5	3.5	101.23	4.76	0.97
	S	4.8 ± 0.4	–	102.09	1.58	0.99	18.9 ± 2.0	–	99.16	3.21	0.99
2,4-D	R1	164.8 ± 24.8	3.3	101.31	1.77	0.97	469.5 ± 28.5	3.9	101.07	4.69	0.97
	R2	461.3 ± 38.1	9.2	99.16	1.79	0.99	1076.6 ± 57.3	9.0	100.01	6.01	0.98
	S	50.1 ± 3.1	–	101.12	1.17	0.98	119.1 ± 9.0	–	103.05	3.51	0.99
Pyraflufe-ethyl	R1	0.41 ± 0.08	5.9	100.04	2.13	0.97	1.05 ± 0.31	1.7	99.33	1.53	0.99
	R2	2.9 ± 0.16	41.4	99.23	1.38	0.99	8.42 ± 1.05	13.8	100.42	2.37	0.98
	S	0.07 ± 0.01	–	100.01	0.68	0.97	0.61 ± 0.06	–	99.66	1.34	0.99

*Data were pooled and fitted using the non-linear regression model: Y = (d)/1+(x/g)^b^, where Y is the is the fresh weight reduction or survival percentage; x (independent variable) the herbicide dose; d is the upper limit; b is the slope of the curve; LD_50_ and GR_50_ expressed as g ai ha^−1^ or g ae ha^−1^, in the case of glyphosate. ^b^RF = Resistance Factor = GR_50_ or LD_50_ of an R biotype/GR_50_ or LD_50_ of the S biotype. ± Standard error of the mean (n = 8)*.

Glyphosate showed low levels of efficiency on S, R1 and R2 biotypes. The three *E. ciliatum* biotypes had LD_50_ values close to the glyphosate field dose (1,080 g ea ha^−1^) used by farmers. The RF values of the R1 and R2 biotypes in respect to the S biotype were 1.1 and 1.0, respectively (Table 3).

The S biotype was very sensitive to flazasulfuron; and the LD_50_ and GR_50_ values the R1 and R2 biotypes, which presented similar profiles of resistance, were close to the field dose of this herbicide (Table 3). The resistance to flazasulfuron in *E. ciliatum* had already been selected in field by showing poor control.

The R1 biotype had low resistance to glufosinate based on both the LD_50_ and GR_50_ parameters, compared to the S biotype. The R2 biotype had LD_50_, GR_50_, and RF values that were higher than the R1 biotype. The LD_50_ of R2 biotype was higher than the field dose confirming its resistance to glufosinate after the recurrent selection (Table 3).

Data associated with survival and growth reduction show that the S biotype was more susceptible to 2,4-D than the R1 and R2 biotypes. The R1 and R2 *E. ciliatum* biotypes were 3.3 and 9.2 more resistant then the S biotype (Table 3), showing that the resistance to 2,4-D increased markedly after the recurrent selection.

The S and R1 biotypes 48 HAT presented visible symptoms with pyraflufen-ethyl, meanwhile, the R2 plants had no presented damage. At 96 HAT, chlorosis and necrosis corona damages became evident in the S biotype and less so in some plants in the R1 biotype. The R2 biotype remained green with little damage in adult leaves. After 1 week, all S plants and only a small number of R1 plants died and all R2 plants survived. Based on the LD_50_ and GR_50_ values, the biotypes R1 and R2 were 1.7 and 13.8 or 5.9 and 41.4 more resistant, respectively, than the S biotype (Table 3).

### Physiological and biochemical studies

#### Diquat

The average total recovery of ^14^C-diquat applied was >93% in both R1 and S biotype. ^14^C-diquat absorption was similar in S and R1 *E. ciliatum* biotypes throughout the measurement period, increasing over time up to 80% at 24 HAT. However, the translocation of ^14^C-diquat from the treated leaf to the rest of the shoot and roots was greater and faster in the S biotype than in the R1 biotype. Thus, we can observe a translocation of 34.9% in the rest of the plant and 18.3% in the root system in the *E. ciliatum* S biotype after 24 HAT. In the biotype R1, translocation was reduced with values of 3.1% in the rest of plant and a negligible amount of ^14^C-diquat in the root system (Table 4).

**Table 4:** ^14^C-diquat absorption and translocation from three to 24 h after treatment (HAT) in R1 and S *Epilobium ciliatum* plants.

**Biotype**	**HAT**	**Absorption(%)^a^**	**Translocation (% absorbed)**^b^		
			**TL**	**RP**	**RS**
S	3	10.3 ± 1.4 E	97.1 ± 2.3 A	2.3 ± 0.4 D	0.6 ± 0.2 C
	6	21.4 ± 3.1 D	81.4 ± 5.9 B	10.2 ± 2.5 C	8.4 ± 1.9 B
	12	49.7 ± 6.8 C	68.3 ± 6.4 C	17.1 ± 3.4 B	14.6 ± 2.1 A
	24	81.2 ± 5.9 A	46.8 ± 3.9 D	34.9 ± 6.8 A	18.3 ± 3.4 A
R1	3	8.4 ± 2.2 E	98.3 ± 0.1 A	1.5 ± 0.3 D	ND
	6	23.2 ± 5.7 D	97.9 ± 1.8 A	1.8 ± 0.9 D	ND
	12	61.3 ± 4.8 B	98.1 ± 1.5 A	1.4 ± 0.4 D	ND
	24	79.2 ± 3.5 A	96.4 ± 3.3 A	3.1 ± 1.1 D	0.5 ± 0.09 C

a *Percentage of ^14^C-diquat absorbed from total applied*.

b *TL, treated leaf; RP, remainder of the plant; RS, root system. Means followed by the same letter per column do not differ by the Tukey test (P < 0.05). ± Standard error (n = 5). ND, no detected*.

#### Glyphosate

**Figure 2:**
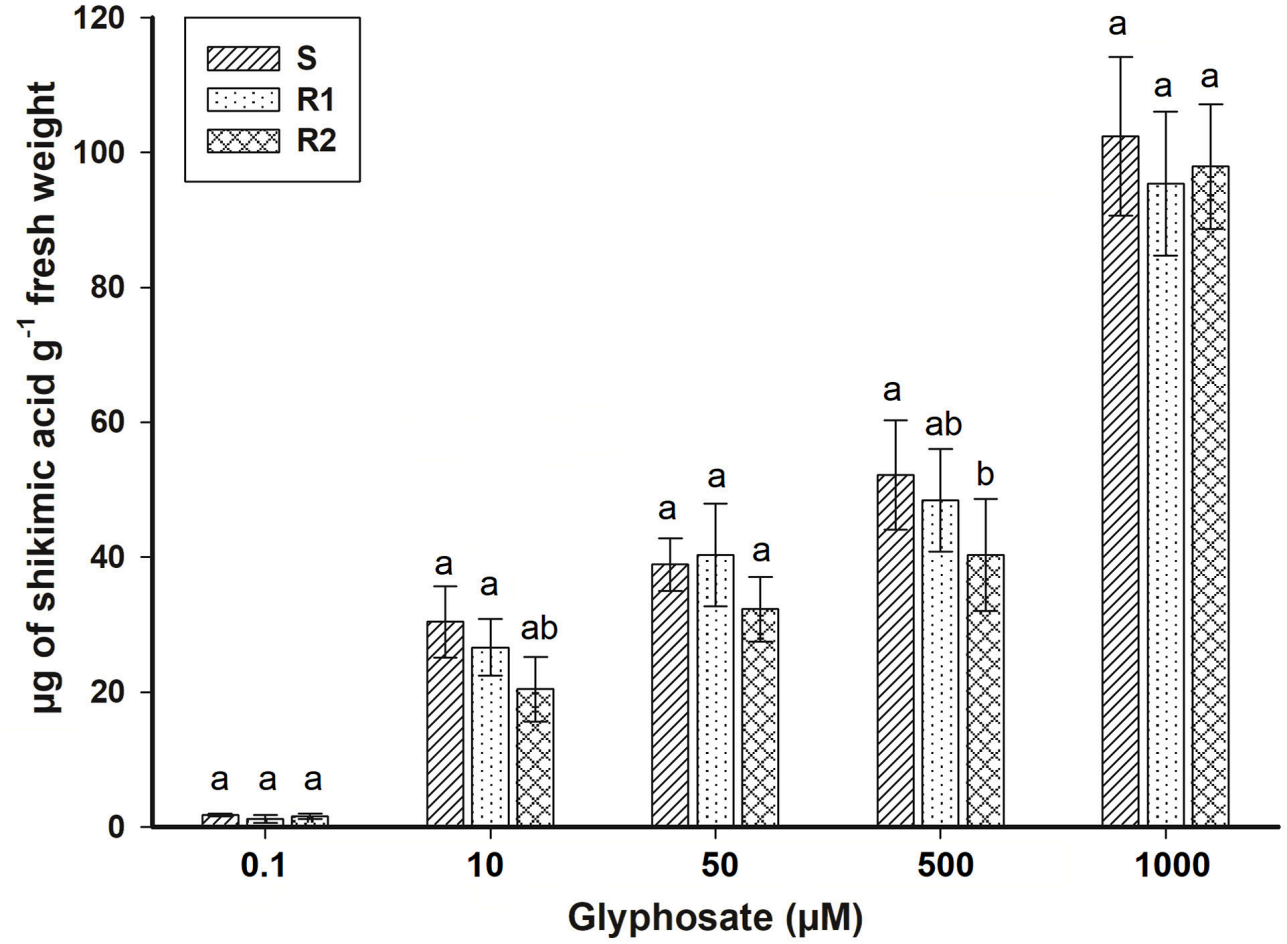
Shikimic acid accumulation in bipyridylium-resistant *Epilobium ciliatum* biotypes at different glyphosate concentrations. Means followed by the same letter do not differ by the Tukey test (*P* < 0.05). Vertical bars are ± standard errors of the mean (*n* = 3).

*Epilobium ciliatum* plants treated with increasing glyphosate concentrations accumulated shikimic acid. However, no differences in shikimate accumulation were observed between the three biotypes (Figure 2). These results are consistent with those obtained previously from the dose-response assays (Table 3).

#### Flazasulfuron

**Figure 3:**
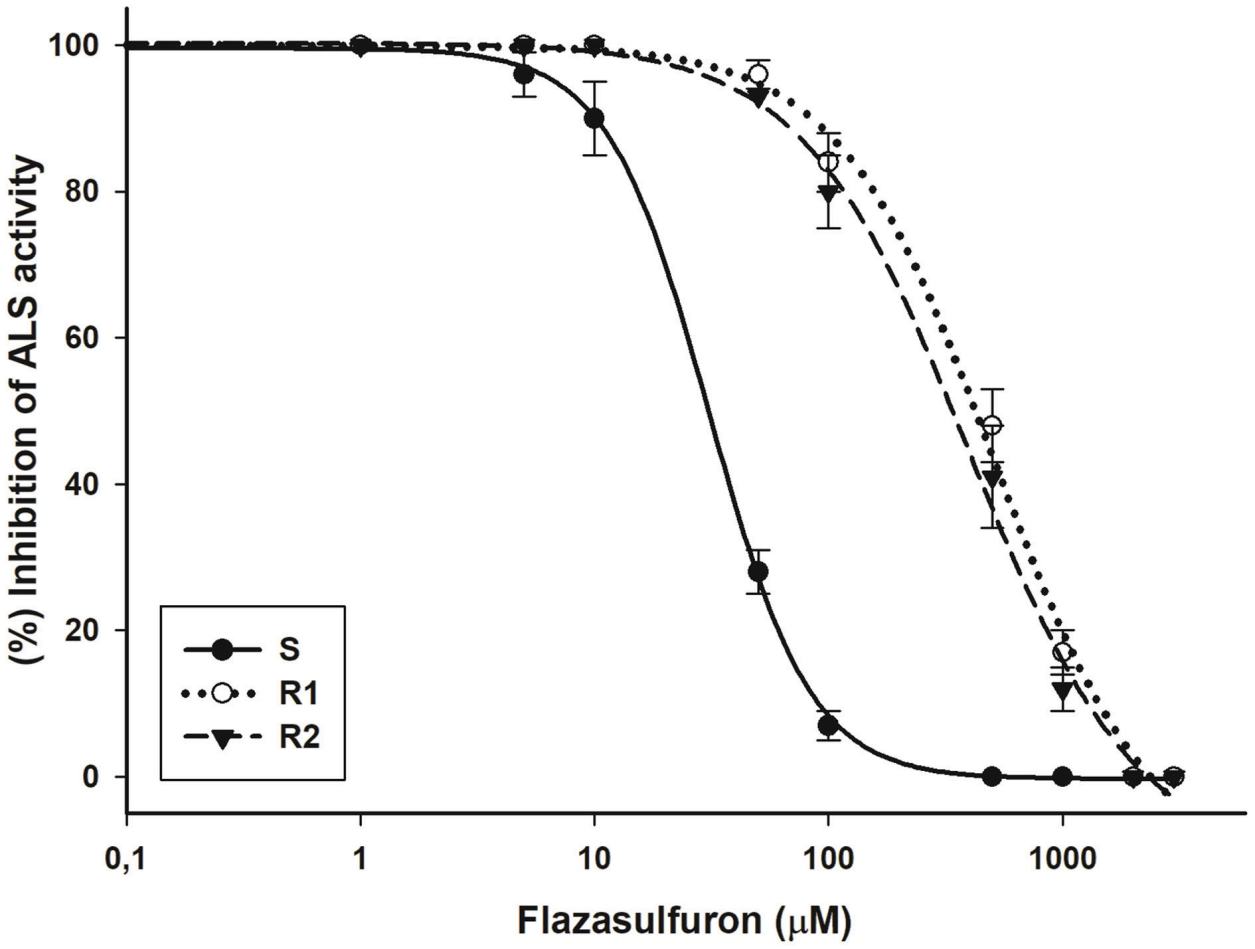
Acetolactate synthase (ALS) enzyme activity in bipyridylium-resistant *Epilobium ciliatum* biotypes determined using flazasulfuron. The equations of log–logistic curves to estimates the I_50_ values are: S: Y= 0.31+{(99.53–0.31)/[1+ (dose/I_50_)^2.00^]}, (R^2^ = 0.99); R1: Y = −3.40+{(99.94+3.40)/[1+(dose/I_50_)^1.30^]}, (R^2^ = 0.99): R2: Y = −1.17+{(100.26+1.17)/[1+(dose/I_50_)^1.22^]}, (R^2^ = 0.99). Vertical bars are ± the standard errors of the mean (*n* = 3).

The inhibition of the ALS enzyme in the R1 and R2 biotypes was similar, and they were 16.4 and 14.9 less susceptible, respectively, than the S biotype (I_50_ = 30.9 μM flazasulfuron) (Figure 3). The specific activity of the ALS enzyme was similar across the three biotypes (318.2, 322.1, and 316.9 nmol acetoin per mg protein per hour for S, R1, and R2, respectively).

#### Glufosinate

**Figure 4:**
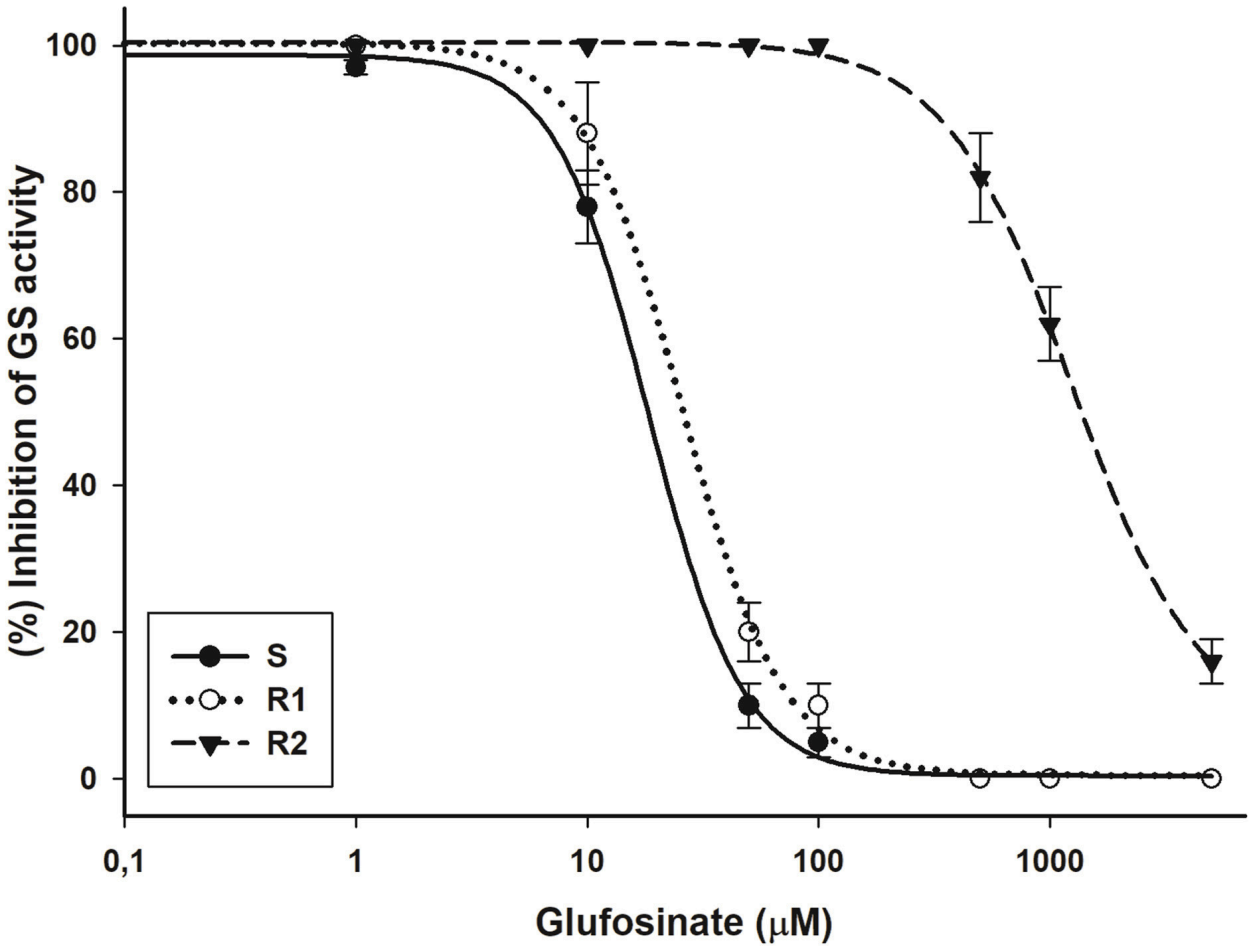
Glutamine synthetase (GS) inhibition by glufosinate in bipyridylium-resistant *Epilobium ciliatum* biotypes. The equations of log–logistic curves to estimates the I_50_ values are: S: Y= 0.39+{(98.64–0.39)/[1+ (dose/I_50_)^2.14^]},(R^2^ = 0.99); R1: Y = 0.50+{(100.30–0.50)/[1+(dose/I_50_)^1.99^]},(R^2^ = 0.99); R2: Y=6.55+{(100.39–6.55)/[1+(dose/I_50_)^1.57^]}, (R^2^ = 0.99). Vertical bars are ± the standard errors of the mean (*n* = 3).

The glufosinate doses required to reduce GS activity by 50% (I_50_) were 18.5, 25.8, and 1252.3 μM for the S, R1, and R2 biotypes, respectively. The RF values indicated that R2 was 67.7-fold more resistant than the S biotype; whereas GS activity was inhibited by glufosinate in a similar way in the S and R1 biotypes (Figure 4).

#### 2,4-D

**Figure 5:**
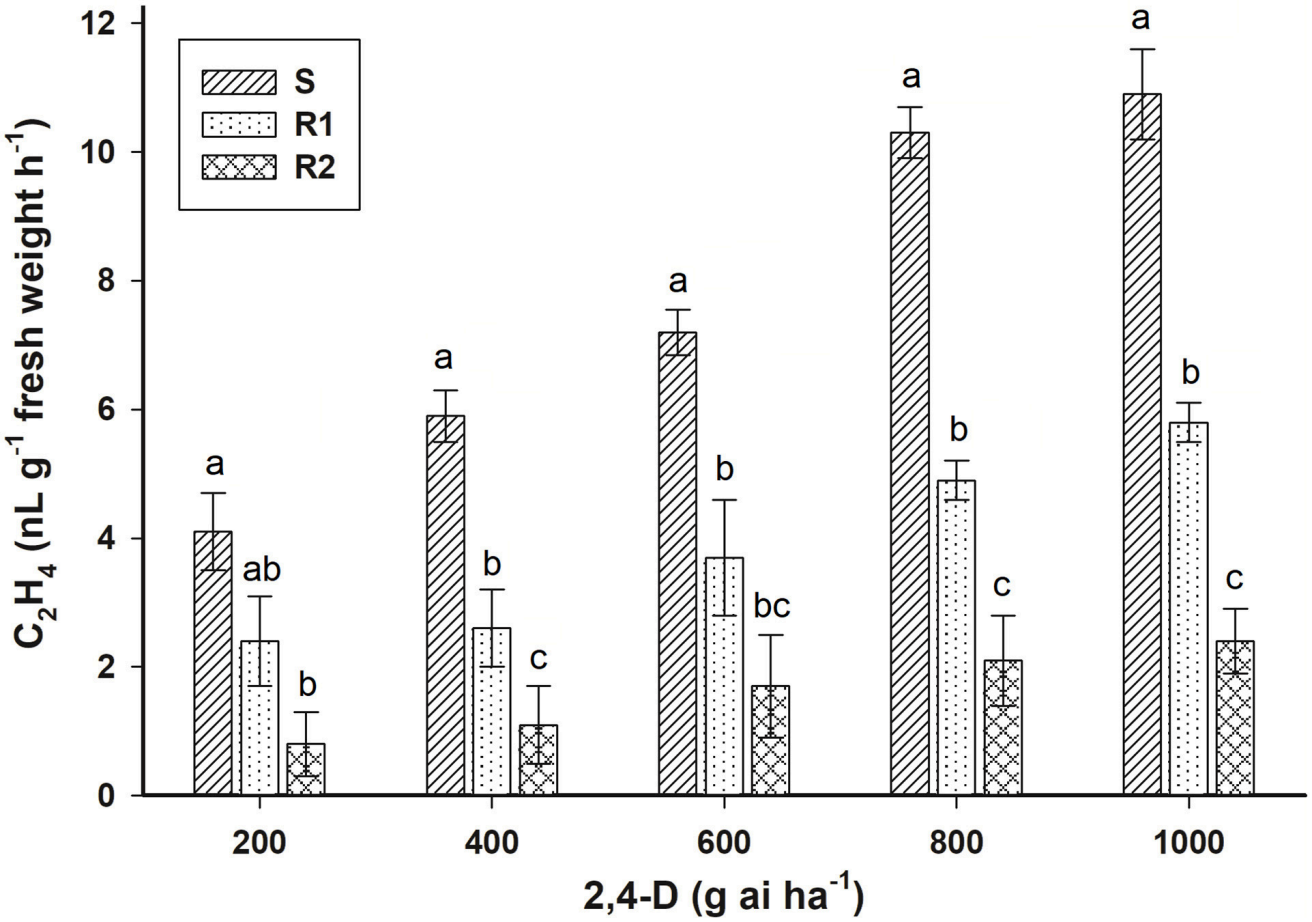
Ethylene accumulation induced by 2,4-D in S, R1 and R2 plants of the bipyridylium-resistant *Epilobium ciliatum* biotypes. Means followed by the same letter do not differ by the Tukey test (*P* < 0.05). Vertical bars are ± the standard errors of the mean (*n* = 5).

The ethylene accumulation in *E. ciliatum* S, R1, and R2 plants responded positively to the dose of herbicide applied (Figure 5). Non-treated plants from the three biotypes had similar ethylene accumulation (0.14 nL g^−1^ fresh weight h^−1^). The results of accumulation of ethylene at 800 g ai ha^−1^ of 2,4-D were 2.0 and 5.0 times smaller in the R1 and R2 biotypes, respectively, than accumulation in the biotype S.

#### Pyraflufen-ethyl

After 100 μM piraflufen-ethyl application, the R2 biotype accumulated significantly less Protox IX than the S and R1 biotypes of *E. ciliatum*. The Protox IX accumulation values were 0.258, 1.420, and 3.062 nmol g^−1^ fresh weight in R2, R1, and S *E. ciliatum* biotypes, respectively.

## Discussion

The olive (*Olea europaea* L.) is one of the oldest fruit trees used by man. At present, this is an innovative and expanding crop in Chile (20,000 ha in 2017), which has been integrated into the new olive growing locations around the globe (Barranco et al., 2017). The use of herbicides is the most widely used tool for Chilean weed control (Valverde, 2007), in some cases, rotating between herbicides with different MOA. However, this practice has lead to the evolution of resistance to multiple herbicides that utilize different resistance mechanisms.

The R1 *E. ciliatum* biotype, which had been treated for consecutive years by bipyridium herbicides (diquat/paraquat), survived at doses higher than those used by the farmer. However, the S biotype, harvested in an area that was never treated by diquat/paraquat, was efficiently controlled at these doses. These results evidence the evolution of resistance of the R1 *E. ciliatum* biotype to bipyridium herbicides. Presently, there have been 67 reports of resistance to paraquat and only 10 to diquat (Heap, 2018). *Epilobium ciliatum* was detected as being resistant to paraquat in Belgium in 1986, but not to diquat (Bulcke et al., 1987). Our results demonstrate cross-resistance to both herbicides. Applications at the tillering stage were much less efficient than those applied at the rosette stage, which indicates that these herbicides need to be applied at the early stages for greater efficiency.

The plant resistance to bipyridyliums may be due to protective enzymes, which minimizes reactive oxygen species, although, resistance to these herbicides is usually due to reduced movement of the herbicide into plants (Preston, 1994; Hawkes, 2014; Moretti and Hanson, 2017). The results with ^14^C-diquat demonstrated that both biotypes, S and R1, have similar capacities for herbicide absorption during the first 24 HAT. However, the reduced translocation of ^14^C-diquat observed within the leaf in the R1 biotype in the basipetal direction, compared to the highest and most rapid translocation found in the S biotype, indicates that this physiological alteration contributes to resistance. The lack of translocation was characterized as being the mechanism responsible for resistance to paraquat in species of the genera *Conyza* (Kato and Okuda, 1983; Fuerst et al., 1985) and *Hordeum* (Powles, 1986; Turcker and Powles, 1991). In *Lolium perenne*, translocation of paraquat from the treated leaf was lower in a resistant biotype than in a susceptible one (Brunharo and Hanson, 2017). This reduced translocation was the result of a higher transport of the herbicide into the vacuole, which could also be considered a resistance mechanism.

Chilean farmers have always complained about the poor control of *E. ciliatum* with glyphosate, even if used at high doses. The similar susceptibility patterns found in the S, R1, and R2 biotypes, according to the dose-response and shikimate accumulation results, allow us to conclude that *E. ciliatum* has natural tolerance to glyphosate, which can be explained by mechanisms of the non-target site type (Bracamonte et al., 2018). The lack of ^14^C-glyphosate absorption and translocation, and in other cases, the glyphosate metabolism into non-toxic compound, has been characterized as the mechanisms responsible for the tolerance to this herbicide (Cruz-Hipolito et al., 2011; Ribeiro et al., 2015).

The R1 and R2 *E. ciliatum* biotypes presented LD_50_ values of flazasulfuron higher than the field dose, which supports that the resistance to this herbicide was already selected for in the field. The similar inhibition of the ALS enzyme in R1 and R2 biotypes suggests that a mutation in the target site may be responsible for the resistance found in both R biotypes. There are 159 weeds species that are resistant to the ALS inhibiting herbicides (Heap, 2018), but only *Senecio vulgaris* and *Cyperus brevifolius* were detected being resistant to flazasulfuron (Okuno et al., 2015; Délye et al., 2016). In most cases, the resistance to these herbicides is due to mutation(s) in different position(s) of the ALS gene, but due to the action of specific enzymes, herbicide metabolic processes may also be involved (Yu and Powles, 2014).

Resistance to glufosinate, 2,4-D and pyraflufen-ethyl in the bipyridylium-resistant *E. ciliatum* (biotype R1), as mentioned above, was not clear in our first set of dose-response assays, nor has been confirmed by the farmers. However, the F_3_ progeny (R2 biotype) of *E. ciliatum* displayed great resistance after the recurrent selections, with LD_50_ values that were above field doses. Obviously, our experimental conditions were extreme by selecting only the most resistant R1 individuals that gave origin to the R2 biotype, and by suppressing the genetic variability existing in natural conditions (Neve and Powles, 2005). Therefore, this condition of multiple resistance will take in appear more than three cropping season or selections, but due to the signs of control failure of *E. ciliatum* in field, this situation could be a reality in Chilean olive orchards, if alternatives to weed management, other than herbicides, are not included. Physiological and biochemical tests suggested the primary mechanisms that are likely involved in the multiple resistance to these herbicides in the R *E. ciliatum* biotypes.

The similar susceptibility to glufosinate in the R1 and S biotypes observed in the dose-response and GS activity assays, suggests that the R1 biotype was sensitive to this herbicide, and resistance in R2 was due to recurrent selection. These results suggest there may be a mutation in the GS gene of the progeny R2 biotype. To date, there are only four glufosinate-resistant weed species confirmed (Avila-Garcia and Mallory-Smith, 2011; Ghanizadeh et al., 2015; Fernandez et al., 2017; Jalaludin et al., 2017), but only in *Lolium perenne* ssp. *multiflorum* from Oregon (USA), a mutation was found in the GS gene endowing resistance to this herbicide (Avila-Garcia et al., 2012).

Plants treated with auxin herbicides, such as 2,4-D, show a fast accumulation of ethylene that is a function of the greater or lesser toxicity of these herbicides in treated plants (Mithila et al., 2011; Busi et al., 2017). The results obtained in our experiments indicated that 2,4-D was not reaching its target, a nuclear auxin receptor (F-box family), as determined by the ethylene synthesis pathway (ACC synthase) in the R2 biotype. There are 31 weeds with resistance to fenoxy-carboxylic acids (16 to 2,4-D) worldwide (Heap, 2018), and in the majority of these, non-target site resistance acts as the mechanisms (Busi et al., 2017; Torra et al., 2017). Among those, impaired translocation in resistant plants, compared to susceptible plants, is related to reduced ethylene production because 2,4-D is not reaching its nuclear target, such as *Papaver rhoeas* (Rey-Caballero et al., 2016).

Pyraflufen-ethyl, a novel inhibitor of Protox IX, is an effective herbicide in early post-emergence acting quickly on broad-leaved weeds at very low rates. The accumulation of the Protox IX can be used to determinate the efficacy of PPO inhibiting herbicides, where the greater accumulation of this enzyme corresponding to susceptible plants (Dayan et al., 2015). The accumulation of the Protox IX in plants treated with piraflufen-ethyl was lower in R2 plants than in R1 and S plants, confirming the higher resistance of the R2 biotype. These results support that the S and R1 biotypes were susceptible to pyraflufen-ethyl and that the R2 biotype evolved resistance after three recurrent selections. *Ambrosia artemisiifolia* was documented as the first resistance case to PPO inhibiting herbicides in 2004 (Rousonelos et al., 2012). To date, 13 cases of PPO-resistant weeds have been documented, 10 belonging to dicotyledonous weeds and three to monocotyledonous (Heap, 2018). The levels of resistance are quite variable between the different species and the herbicides tested (Dayan et al., 2014). The genus *Amaranthus* (*A. hybridus, A. palmeri, A. retroflexus*, and *A. tuberculatus*) detected in several cropping areas in the USA that has multiple resistance to glyphosate, ALS and PPO has been studied in depth (Legleiter and Bradley, 2008; Salas et al., 2016). Molecular studies have shown that a mutation in the PPO gene at position 98 is the most likely resistance mechanism involved in *A. artemisiifolia* (Rousonelos et al., 2012) and *A. palmeri* (Giacomini et al., 2017).

## Conclusion

Based on our results, we can conclude that *E. ciliatum* harvested directly in field was resistant to bipyridyliums and flazasulfuron. Glyphosate is not an alternative in controlling *E. ciliatum* due to its innate tolerance to this herbicide. The condition of multiple resistance to five MOAs (ALS, GS, PPO, PSI inhibitors, and synthetic auxin) could occur in next cropping seasons, as demonstrated by the recurrent selection, if alternatives to weed management, other than herbicides, are not included.

## Author contributions

RD: idea and designed of the research. BT, RA-dlC, and AR-D performed the experiments. BT, RA-dlC, EA, JT, JD-V, HC-H and AR-D interpreted and analyzed the raw data. BT, RA-dlC, EA, JT, JD-V, HC-H, AR-D and RD wrote and approved the manuscript.

### Conflict of interest statement

The handling editor is currently co-organizing a Research Topic with one of the authors RD, and confirms the absence of any other collaboration. The other authors declare that the research was conducted in the absence of any commercial or financial relationships that could be construed as a potential conflict of interest.
